# Beyond the brain circuit: a biopsychosocial integrative review of addiction neuroscience, epigenetic mechanisms, and multidisciplinary treatment

**DOI:** 10.3389/fpsyt.2026.1887517

**Published:** 2026-09-04

**Authors:** Yunus İçer

**Affiliations:** Isparta Provincial Directorate of Youth and Sports, Ministry of Youth and Sports, Isparta, Türkiye

**Keywords:** addiction, attachment, epigenetics, integrative review, multidisciplinary treatment, neuromodulation, neuroscience

## Abstract

This integrative review examines the neurobiological basis of addiction and neuromodulation-based treatment approaches in light of current literature, while proposing an integrative framework that extends beyond purely biological explanations. The neuroscience of addiction is addressed within Koob and Volkow’s three-stage neurocircuit model (binge/intoxication, withdrawal/negative affect, and preoccupation/craving), encompassing neuroadaptations in reward, stress, and executive control circuits, neuroimaging findings, genetic and epigenetic mechanisms, and neuromodulation-based treatment approaches. A central argument of this review is that addiction is not solely a brain circuit disorder but also a disorder of social connection and meaning: trauma, early adversity, insecure attachment, and social isolation constitute both neurobiological triggers and perpetuating factors of addiction, leaving epigenetic traces on reward and stress circuits that interact with genetic vulnerability. Genome-wide association studies emphasize polygenic risk architecture, while epigenetic mechanisms — particularly DNA methylation and histone modifications — translate environmental exposures into lasting neuroadaptations. Critically, the distinction between state and trait epigenetics must be carefully considered when interpreting cross-sectional findings, as longitudinal designs are required to establish translational clinical validity. Neuromodulation techniques, including repetitive transcranial magnetic stimulation (rTMS) and transcranial direct current stimulation (tDCS), have demonstrated promising results in reducing craving and relapse risk; however, clinical application requires careful patient selection, standardized multi-session protocols, and integration with pharmacotherapy and psychotherapy. This review proposes a three-layer multidisciplinary treatment framework — biological, psychological, and social-relational — in which neuromodulation serves as one component of a comprehensive approach that targets the neurobiological, psychological, and relational dimensions of addiction simultaneously.

## Introduction

Addiction is one of the most pressing public health challenges of our time, associated with high rates of morbidity, mortality, and psychosocial burden worldwide. Over the past three decades, advances in neuroscience have fundamentally reshaped how addiction is understood: rather than a failure of willpower or a moral deficit, it is now widely conceptualized as a chronic, relapsing brain disorder characterized by persistent neuroadaptations in reward, stress, and executive control circuits (1, 2). This neurobiological framework has been instrumental in reducing stigma, informing pharmacological interventions, and generating a robust body of translational research.

Yet the Brain Disease Model of Addiction (BDMA), while scientifically valuable, carries inherent limitations when applied in isolation. A growing body of evidence indicates that neurobiological vulnerability cannot be fully understood apart from the relational, developmental, and social contexts in which it emerges. Adverse childhood experiences leave epigenetic imprints on reward and stress circuits (3, 4); insecure attachment styles predict earlier onset of substance use (5); and social isolation shares a neurobiological substrate with opioid dependence through the endogenous opioid system (6). These findings collectively suggest that addiction is not solely a disorder of brain circuits, but also a disorder of social connection and meaning — and that treatment frameworks must reflect this complexity.

This integrative framework does not emerge in a conceptual vacuum. The proposal that health and illness must be understood through the joint operation of biological, psychological, and social factors was foundationally articulated by Engel (7) in his critique of the biomedical model, and remains the conceptual ancestor of any claim that addiction is not solely a brain disorder. More recently, the Research Domain Criteria (RDoC) framework, and its addiction-specific adaptation, the Alcohol and Addiction RDoC (AARDoC), has proposed a transdiagnostic, dimensional mapping of negative emotionality, incentive salience, and executive function that closely parallels the three-stage neurocircuit model discussed below (8). Empirical work applying the RDoC framework to substance use has begun to substantiate this dimensional logic: in a structural neuroimaging study of polysubstance use, reduced limbic gray matter volume was found to predict substance use severity indirectly, through its association with emotional dysregulation (anxiety and depression) rather than through a direct structural pathway alone (9) — a finding consistent with this review’s broader argument that biological vulnerability and psychosocial/emotional adversity are mechanistically intertwined rather than separable.

The present integrative review is guided by this central argument. Rather than treating neurobiological, epigenetic, and psychosocial dimensions as parallel but separate domains, we propose that they form an integrated explanatory framework in which each layer informs the others. Among the biological interventions available for addiction, neuromodulation occupies a distinctive position: unlike pharmacotherapy, whose mechanism of action is systemic and neurochemical, neuromodulation targets a circuit-level deficit — DLPFC hypofunction — that can be directly visualized through neuroimaging and whose modulation can be measured session by session. This makes it a uniquely tractable bridge between the circuit-level evidence reviewed above and a concrete, individualizable treatment target. Neuromodulation techniques — including repetitive transcranial magnetic stimulation (rTMS) and transcranial direct current stimulation (tDCS) — are examined within this framework not as standalone interventions, but as one biological component of a broader, three-layer multidisciplinary treatment model. In doing so, this review aims to extend beyond purely biological accounts of addiction and contribute a clinically actionable framework that integrates recent advances in neuroscience, epigenetics, and relational psychology.

To this end, the review pursues four specific aims: (1) to synthesize current neurobiological evidence on the three-stage addiction cycle and its associated circuit-level disruptions; (2) to critically examine genetic and epigenetic mechanisms, with particular attention to the state/trait distinction and the role of early adversity; (3) to evaluate the clinical evidence base for neuromodulation techniques, including patient selection criteria, protocol considerations, and combination with psychotherapy; and (4) to propose and contextualize a three-layer integrative treatment framework — biological, psychological, and social-relational — in relation to existing clinical models.

## Method

This integrative review was conducted through systematic keyword searches across two major databases: PubMed and Google Scholar. The search covered the period 1993–2026. The lower bound reflects the deliberate inclusion of a small number of foundational theoretical works that remain the empirical and conceptual basis for contemporary addiction research; for example, the incentive-sensitization theory (10) is still the dominant explanatory framework for reward-circuit dysfunction cited throughout the neurobiological literature reviewed here, and is retained for this reason rather than for historical completeness. The great majority of the literature is nonetheless recent: approximately 67% of cited sources were published between 2021 and 2026, reflecting a strong emphasis on current evidence.

Search terms included, among others: *addiction AND neurobiology*; *substance use disorder AND epigenetics*; *adverse childhood experiences AND substance use*; *neuromodulation AND addiction*; *transcranial magnetic stimulation AND substance use disorder*; *tDCS AND craving*; *attachment AND substance use disorder*; *GWAS AND substance use disorder*; and *biopsychosocial model AND addiction*.

Article selection followed the iterative, topic-driven search process typical of integrative reviews (11): searches were conducted per subtopic using the keyword combinations above, primarily via Google Scholar, with priority given to 2022–2024 publications and extension to earlier foundational work where recent coverage was limited.

Inclusion criteria encompassed peer-reviewed empirical studies, systematic reviews, meta-analyses, and theoretical articles published in English. Following the integrative review methodology (11, 12), studies from diverse methodological traditions were included to enable synthesis across neurobiological, epigenetic, and psychosocial domains. Case reports, studies focusing exclusively on pharmacological interventions without broader treatment context, and publications without full-text access were excluded.

The integrative review approach was selected because the review’s central aim — proposing a unified three-layer treatment framework — requires the synthesis of findings across heterogeneous methodologies and disciplines (11). Unlike systematic reviews, integrative reviews are particularly suited to the development of new conceptual frameworks from existing evidence, and to the reformulation of established models in light of emerging findings across multiple fields (12).

## Neurobiological foundations of addiction: reward, stress, and executive control circuits

The neurobiological understanding of addiction has undergone a substantial transformation over the past three decades. Contemporary models conceptualize addiction not as a static brain state, but as a dynamic, progressive disorder driven by cumulative neuroadaptations across three functionally distinct yet deeply interconnected circuits: the reward circuit, the stress circuit, and the executive control circuit. The most influential integrative account of these changes is the three-stage neurocircuit model proposed by Koob and Volkow (1), which frames addiction as a cyclical process moving through binge/intoxication, withdrawal/negative affect, and preoccupation/craving. Each stage is associated with specific neurobiological disruptions and, critically for the present review, with distinct psychological and relational manifestations that have direct implications for clinical intervention.

### The reward circuit and incentive-sensitization

The mesocorticolimbic dopamine system — comprising the ventral tegmental area (VTA), nucleus accumbens (NAc), and prefrontal cortex (PFC) — constitutes the primary neural substrate of reward processing and motivational salience (1). Under normal conditions, this system encodes the value of natural rewards such as food, social bonding, and sexual pleasure. Addictive substances hijack this circuitry by producing dopamine release that is substantially stronger, faster, and more sustained than any natural reward can generate, progressively recalibrating what the brain treats as motivationally significant (13).

The incentive-sensitization theory (10, 13) provides a critical conceptual distinction here: dopamine is not primarily a *pleasure* signal, but a *wanting* signal. As substance exposure repeats, the mesolimbic system becomes sensitized to substance-related cues, generating increasingly intense motivational drive (*wanting*) even as the hedonic experience of the substance (*liking*) remains stable or declines due to tolerance. This dissociation — compulsive wanting without commensurate pleasure — is a hallmark of the binge/intoxication stage and helps explain why individuals continue substance use despite diminishing returns. From a clinical standpoint, this neurobiological process maps onto the psychological experience of craving as an intrusive, ego-dystonic urge that feels disconnected from genuine enjoyment or choice.

### Habit formation and the transition to compulsive use

A defining feature of addiction is the progressive transition from goal-directed to habitual and ultimately compulsive substance-seeking. Everitt and Robbins (14) describe this as a shift in the locus of behavioral control: early substance use is governed by the ventral striatum and prefrontal cortex, which support flexible, outcome-sensitive decision-making. With repeated use, control migrates toward the dorsolateral striatum and caudate-putamen, brain regions associated with stimulus-response (S-R) habit learning and behavioral automaticity.

This neurobiological transition has profound clinical implications. Once substance-seeking has become habitual, it is no longer primarily responsive to reasoning, consequences, or motivation — it is triggered automatically by environmental cues, emotional states, or interpersonal contexts. This is why psychoeducation and motivational approaches alone are often insufficient in later-stage addiction: the behavior has, in a meaningful neurobiological sense, become decoupled from conscious deliberation. Intervention at this stage requires approaches that directly address automaticity — including behavioral rehearsal, cue exposure with response prevention, and neuromodulation targeting prefrontal-striatal connectivity.

### The stress circuit, extended amygdala, and negative reinforcement

The withdrawal/negative affect stage is governed primarily by the extended amygdala — a network encompassing the central nucleus of the amygdala, the bed nucleus of the stria terminalis, and the shell of the nucleus accumbens (1). During this stage, the brain’s anti-reward system becomes chronically activated, generating persistent negative emotional states including anxiety, dysphoria, irritability, and anhedonia. Critically, repeated substance use progressively lowers the hedonic baseline, meaning that the individual’s emotional equilibrium becomes dependent on substance use to maintain.

This mechanism represents a fundamental shift in the motivational architecture of addiction: from positive reinforcement (using to feel good) to negative reinforcement (using to avoid feeling bad). The allostatic load accumulated in stress circuits means that abstinence is not experienced as a neutral state but as an aversive one (1). This explains why relapse risk is highest not only during periods of craving or cue exposure, but during periods of sustained negative affect, interpersonal conflict, and social disconnection — conditions that directly activate the extended amygdala.

### Executive control and prefrontal dysfunction

The preoccupation/craving stage is characterized by dysfunction in the prefrontal cortex (PFC), particularly the dorsolateral prefrontal cortex (DLPFC) and the anterior cingulate cortex (ACC). These regions are responsible for executive functions including impulse inhibition, decision-making, working memory, and cognitive flexibility (15). In individuals with addiction, chronic downregulation of PFC activity results in impaired capacity to override drug-seeking impulses, weigh long-term consequences against immediate urges, and disengage from substance-related thought patterns.

Neuroimaging meta-analyses consistently document reduced activation in the DLPFC and inferior frontal gyrus during response inhibition tasks in substance use disorders (16), providing convergent evidence that executive control deficits reflect underlying circuit-level disruptions rather than motivational deficits or treatment non-compliance. This finding directly informs the rationale for DLPFC-targeted neuromodulation.

### The three-stage cycle: an integrated clinical summary

**Figure 1:**
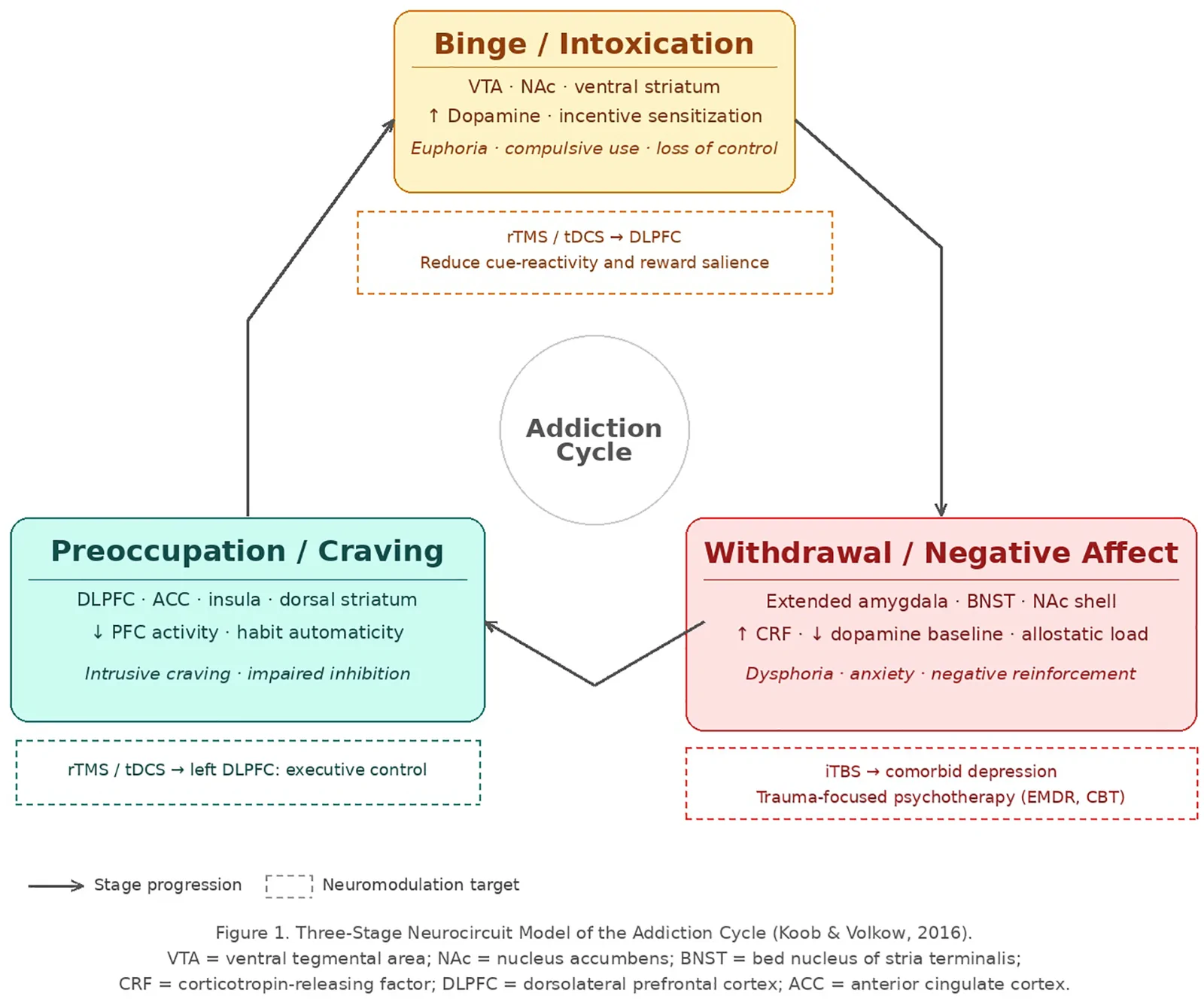
Three-stage neurocircuit model of the addiction cycle (1). VTA, ventral tegmental area; NAc, nucleus accumbens; BNST, bed nucleus of the stria terminalis; CRF, corticotropin-releasing factor; DLPFC, dorslateral prefrontal cortex.

Taken together, these three circuits operate not as independent systems but as a self-reinforcing cycle. As illustrated in **Figure 1**, each stage feeds into the next: binge/intoxication produces neuroadaptations that deepen withdrawal severity; withdrawal-driven negative affect intensifies preoccupation and craving; and impaired executive control reduces the capacity to interrupt the cycle. With each repetition, neuroadaptations consolidate and behavioral flexibility narrows (1).

## Stuctural and functional brain changes in addiction: neuroimaging evidence

If the previous section established the theoretical architecture of addiction neuroscience, neuroimaging research provides its empirical foundation. Techniques including functional magnetic resonance imaging (fMRI), positron emission tomography (PET), and structural MRI (sMRI) have made it possible to observe the circuit-level disruptions described by Koob and Volkow’s model directly in living human brains — across substance types, clinical stages, and treatment conditions.

### Prefrontal hypofunction: the neural correlate of lost control

Among the most replicated findings in addiction neuroimaging is reduced activity in the prefrontal cortex, particularly the dorsolateral prefrontal cortex (DLPFC) and inferior frontal gyrus, during tasks requiring response inhibition (15, 16). Meta-analytic evidence consistently demonstrates that individuals with substance use disorders show significantly less activation in these regions when attempting to suppress behavioral responses — a neural signature that maps directly onto the clinical experience of impaired impulse control.

Resting-state studies document reduced baseline DLPFC activity in individuals with addiction, suggesting a chronic deficit in the neural resources available for self-regulation rather than a purely situational impairment (17). This implies that cognitive-behavioral strategies requiring active prefrontal engagement may be working against a neurobiological headwind, particularly in earlier stages of treatment, and provides the primary rationale for DLPFC-targeted neuromodulation as a biological augmentation strategy.

### Striatal hyperreactivity: when the brain stops responding to life

Complementing prefrontal hypofunction, neuroimaging studies consistently document hyperreactivity in striatal regions — particularly the nucleus accumbens and dorsal striatum — in response to substance-related cues (17). A neuroimaging study of cocaine addiction illustrates this dual process: participants showed *decreased* ventral striatal activation during a standard monetary reward task but *increased* activation in the same region when exposed to substance-related cues (18). This dissociation provides a neurobiological account of the anhedonia and loss of interest in previously meaningful activities that clinicians observe in individuals with addiction.

### Network-level disruptions: default mode and salience networks

Addiction-related changes extend beyond discrete brain regions to encompass large-scale functional networks. Multilevel kernel density meta-analyses have identified consistent functional alterations across alcohol, cocaine, nicotine, and opioid use disorders in regions including the insula, anterior cingulate cortex (ACC), and DLPFC — nodes central to both the Default Mode Network (DMN) and the Salience Network (SN) (19). The insula’s role in interoceptive awareness is particularly clinically relevant: insula dysfunction contributes to impaired recognition of physiological craving signals and difficulty distinguishing emotional distress from substance urges, supporting the clinical utility of body-based and mindfulness interventions.

### Structural changes and the evidence for neuroplasticity

Chronic substance use is associated with measurable structural changes, including reductions in gray matter density in the prefrontal cortex, insula, and striatum (16), and significant hippocampal volume reductions in alcohol use disorder alongside volumetric alterations in reward-related structures (20). Crucially, evidence from longitudinal abstinence studies indicates that sustained recovery is associated with partial normalization of gray matter volume and functional connectivity in prefrontal and striatal regions (16) — the neuroplastic potential that makes treatment meaningful.

### Methodological considerations and clinical translation

Most neuroimaging studies employ cross-sectional designs, leaving open whether observed brain differences predate substance use or result from it (17). Rather than serving as diagnostic biomarkers, these findings are best understood as a framework for hypothesis-driven treatment planning. Critically, neuroimaging also reveals what biology alone cannot explain: the same prefrontal hypofunction that predicts poor impulse control is also shaped by trauma history, attachment disruption, and social adversity — mechanisms that are epigenetic in nature.

## Genetic and epigenetic mechanisms: from biological vulnerability to relational history

The neurobiological disruptions documented in the preceding sections do not emerge uniformly across individuals exposed to the same substances or social environments. Understanding genetic and epigenetic mechanisms is essential not because they offer deterministic predictions — they do not — but because they illuminate *why* certain individuals are more vulnerable, *when* in development that vulnerability crystallizes, and *through what biological pathways* relational adversity becomes neurobiological risk.

### Heritability and the limits of genetic determinism

Family, twin, and adoption studies consistently demonstrate significant familial clustering in substance use disorders, with heritability estimates ranging from 40% to 60% across substance types (21–23). However, heritability is a population-level statistical estimate, not a biological blueprint: it describes the proportion of variance attributable to genetic differences under specific environmental conditions. A heritability of 50% does not mean that half of an individual’s addiction risk is fixed at birth; it means that in the studied population, roughly half of the observed variation was associated with genetic variation. This framing shifts the question from *whether* genes matter to *under what conditions* genetic risk is expressed — a question that points directly toward environmental and developmental intervention.

### GWAS findings and polygenic architecture

Genome-wide association studies (GWAS) confirm that addiction risk is broadly polygenic: shaped by the cumulative effect of many common variants, each with small individual effect sizes, converging across multiple biological systems (24–26). Specific variants such as *ADH1B* and *ALDH2* have been identified for alcohol metabolism (23), but these represent exceptions to the general pattern of diffuse, system-level genetic influence. A methodologically important distinction has also emerged: the genetic architecture of *substance use* and *substance use disorder* are highly correlated but not identical phenotypes (25), with direct implications for biomarker development and intervention targeting.

### Missing heritability and gene–environment interaction

A persistent gap separates these two lines of evidence: heritability estimates derived from family and twin studies (40–60%) substantially exceed the proportion of variance explained by the specific genetic variants that GWAS have so far identified, a discrepancy known as the missing heritability problem (27). For substance use disorders specifically, this gap is pronounced: SNP-based heritability estimates from GWAS typically range from only 1% to 28%, well short of the roughly 50% heritability estimated from twin and family studies, and polygenic scores built from current GWAS data explain only about 2–10% of the variance in liability — far too little, on their own, to be clinically useful risk predictors (28). Several non-mutually-exclusive explanations have been proposed, including the aggregate contribution of very-low-effect common variants below current detection thresholds, rare variants poorly captured by standard GWAS arrays, and structural or epigenomic variation not reducible to single-nucleotide polymorphisms (28). It is worth noting that this gap does not appear to reflect a systematic overestimation of heritability by twin studies themselves: sibling-based designs that avoid the assumptions unique to twin research have produced heritability estimates for drug abuse, alcohol use disorder, and related externalizing behavior that closely match those from twins, suggesting the “missing” variance genuinely lies in the genome rather than in a flawed method (29). Phenotypic heterogeneity across studies, limited ancestral diversity in GWAS samples, and high rates of psychiatric comorbidity further complicate the translation of genetic findings for alcohol use disorder specifically into clinically actionable risk estimates (30). Critically, part of this gap is likely attributable not to undiscovered genetic variants but to gene–environment interaction (G×E): the same genetic variant can confer markedly different risk depending on the environmental context in which it is expressed, such that genetic vulnerability alone, measured independently of developmental and relational exposure, systematically understates true risk. A systematic review of 44 candidate-gene studies in substance use found that 38 reported at least one significant G×E effect, with the large majority (roughly 90%) following a “diathesis-stress” pattern: carriers of a risk variant (in genes such as the serotonin transporter, MAOA, OPRM1, and CRHR1) showed elevated substance-use risk mainly when exposed to childhood adversity or chronic stress, while the same variant conferred little added risk in a low-adversity environment (31). This pattern is not limited to substance use: a genetically informative twin study of gaming addiction similarly found that genetic influence on addictive behavior varied as a function of perceived stress, consistent with a diathesis-stress rather than a purely additive model (32). Taken together, this evidence indicates that treating genetic risk as environment-independent understates true vulnerability for individuals facing high adversity while overstating it for those in protective environments, and that gene-by-environment designs are needed to capture this interplay accurately (33). This G×E framing provides the conceptual bridge to the epigenetic mechanisms discussed next, which describe one of the principal biological pathways through which environmental exposure comes to modify genetically encoded risk.

### Epigenetic mechanisms: where environment becomes biology

Epigenetics refers to heritable changes in gene expression that do not involve alterations to the DNA sequence itself (4). Three mechanisms function together as a translation layer between environmental exposures and neurobiological outcomes: DNA methylation, which typically silences gene expression by blocking transcriptional access to promoter regions; histone modifications, which alter chromatin packing and thereby regulate which genes are accessible for transcription; and non-coding RNA regulation, which fine-tunes gene expression post-transcriptionally (4, 21, 34). In the context of addiction, epigenetic processes are the biological mechanism through which chronic stress, trauma, and relational adversity leave lasting imprints on reward sensitivity, stress reactivity, and executive control circuits. Consequently, the circuits that addiction later disrupts are not neutral starting points: they are already shaped by the individual’s developmental and relational history prior to first substance exposure, and this prior shaping determines how those circuits subsequently respond to drug-induced neuroadaptation.

### Adverse childhood experiences and developmental susceptibility

Broekhof et al. (3), in a large community-based cohort study following 8,199 adolescents over 12–14 years, found that exposure to any adverse childhood experience (ACE) was associated with a 4.3-fold increase in the likelihood of developing a substance use disorder in adulthood. Meadows et al. (35) demonstrated that cumulative ACE burden significantly accelerated the age of first substance use. Tuncay et al. (5) extended this evidence to a Turkish clinical sample, demonstrating that insecure attachment styles predicted earlier onset of substance use — confirming that attachment patterns are epigenetically encoded, shaping the stress and reward circuit sensitivity that underlies addiction vulnerability. As Clements et al. (36) synthesized within a social bonding framework, healthy early attachment is associated with reduced addiction risk, and social connection within treatment programs actively supports recovery. These findings position relational history as a major, but not exclusive, contributor to epigenetic risk: addiction-related epigenetic vulnerability is multifactorial, arising from the joint and interacting influence of relational adversity, direct substance exposure, and independent genetic and environmental factors. Furthermore, attachment patterns should not be understood merely as static imprints left by the past; converging evidence suggests that ongoing attachment quality continues to shape epigenetic regulation of stress and reward circuitry in the present, meaning relational history remains a dynamic, and potentially modifiable, influence rather than a fixed residue.

### The state–trait distinction: a methodological and clinical necessity

State epigenetics refers to transient methylation changes driven by acute substance use, withdrawal, or situational stress. Trait epigenetics refers to relatively stable alterations shaped by early developmental experiences, largely independent of current substance use. Cross-sectional studies cannot reliably distinguish between these two categories (4). Komaki et al. (37) provided important longitudinal data showing that a substantial proportion of methylation sites remain stable over time, while dynamic sites disproportionately overlap with immune and inflammatory pathways sensitive to ongoing environmental stress. This distinction determines whether a measured epigenetic change represents a treatment target (state) or a risk factor to be contextualized (trait), and requires longitudinal designs, multi-tissue comparisons, and integration with genetic risk data to resolve. Importantly, the state–trait distinction should not be read as a claim about fixity: even alterations classified as “trait,” originating in early exposure or trauma, are not necessarily unmodifiable. For precision medicine in addiction, the relevant clinical question is therefore not simply whether a given mark is stable or transient, but how the underlying system’s plasticity can be leveraged to promote reversibility of pathological epigenetic states. This is not merely theoretical: animal studies show that directly reversing specific epigenetic marks in the brain’s reward circuitry can undo drug-related behavioral changes already established by prior drug exposure, demonstrating that even “stable” trait-like epigenetic states remain biologically modifiable (38).

Moreover, the evidence discussed above has centered on DNA methylation; other epigenetic mechanisms — such as chemical modifications to the proteins DNA is wrapped around (histones) — also contribute substantially to the cellular “memory” that underlies trait-like stability in response to early environmental exposure, and should be considered alongside methylation in a complete account of state–trait epigenetic dynamics. These histone-level changes occur in brain regions central to reward processing and are seen following exposure to cocaine, methamphetamine, opioids, alcohol, and nicotine, with some changes persisting for weeks after last use — providing a plausible biological substrate for trait-like vulnerability (38–40). These same mechanisms also appear to be shared, at least in part, with the pathways linking addiction to co-occurring depression (41) — an early illustration of a broader principle that recurs throughout the psychiatric comorbidities of addiction: shared biological vulnerability, rather than parallel but independent conditions.

This shared-vulnerability pattern extends well beyond depression. Attention-deficit/hyperactivity disorder (ADHD) shares substantial genetic risk factors with cannabis use disorder specifically, and individuals with ADHD who carry the highest genetic risk for cannabis use disorder face roughly a 22% chance of developing it, compared with about 1.6% among those with lower genetic risk (42). More broadly, genetic studies show that substance use disorders share their strongest overlap in underlying risk factors with ADHD, major depressive disorder, anxiety, and schizophrenia (43). Bipolar disorder shows a similarly robust association at the clinical level: a systematic review and meta-analysis of mood and substance-related disorders found a sixfold elevated likelihood of broadly defined mood disorder co-occurring with drug dependence (44). At the genetic level, cannabis use disorder risk specifically predicts bipolar disorder that includes psychotic features, but not bipolar disorder without psychosis (43) — suggesting that shared vulnerability may operate through specific symptom profiles (such as psychosis proneness) rather than diagnostic categories as a whole. Psychotic disorders show a related, and partly causal, pattern: genetically informed studies support a causal contribution of cannabis use disorder to schizophrenia risk (43), and at the molecular level, methamphetamine use has been linked to a specific epigenetic change in a gene important for the brain’s inhibitory (GABA-related) neurons, which in turn has been associated with methamphetamine-induced psychosis (Y. 45) — offering a candidate biological mechanism that parallels the depression pathway described above.

Taken together, these findings indicate that the genetic and epigenetic vulnerability underlying addiction is inherently transdiagnostic: the same mechanisms that link substance use disorders to depression and trauma extend, through both shared and substance-specific pathways, to ADHD, bipolar disorder, and psychosis. This has a direct implication for the state–trait framework developed above — comorbidity itself is a variable that must be incorporated into longitudinal, multi-tissue research designs, since a given epigenetic mark’s apparent stability may partly reflect shared vulnerability with a co-occurring psychiatric condition rather than a substance-specific developmental imprint.

### Translational implications: toward dynamic biomarkers

The translational potential of epigenetic research in addiction lies not in static risk prediction but in dynamic treatment monitoring: tracking epigenetic change as a recovery indicator. Multicenter, longitudinal studies integrating neuroimaging, epigenetic, clinical, and relational data will be required (4, 24, 25). For present clinical practice, the key implication is immediate: genetic and epigenetic evidence supports a trauma-informed, developmentally sensitive approach in which the biological consequences of relational adversity are treated with the same seriousness as the neurobiological consequences of substance exposure.

## Neuromodulation techniques in addiction treatment: evidence, protocols, and integration

The circuit-level disruptions documented in preceding sections define not only the neurobiological profile of addiction but also a set of specific, targetable treatment objectives. Neuromodulation techniques address these objectives directly by modulating neural activity in the affected circuits through non-invasive electrical or magnetic stimulation.

### Repetitive transcranial magnetic stimulation

rTMS delivers focused magnetic pulses to cortical targets, transiently modulating neuronal excitability in ways that outlast the stimulation session. The primary target in addiction treatment is the DLPFC, based on its central role in impulse inhibition, working memory, and cue-reactivity regulation (15, 16). A systematic review and meta-analysis of 94 studies by Mehta et al. (46) reported moderate-to-high effect sizes (Hedges’ *g* > 0.5) for craving reduction and decreased substance use across alcohol, tobacco, cocaine, methamphetamine, and opioid use disorders, with multi-session left DLPFC protocols producing the most consistent results. Uygur and Celik (47) found that both right and left DLPFC-targeted high-frequency rTMS effectively reduced alcohol craving, while iTBS showed additional promise in reducing comorbid depressive symptoms and relapse risk.

In terms of patient selection, the evidence most consistently supports rTMS for: (1) individuals with high craving burden who have not responded adequately to first-line pharmacotherapy or psychotherapy; (2) individuals in whom neuroimaging reveals DLPFC hypofunction; and (3) individuals with marked impulsivity and deficient executive function profiles (46). Regarding protocol parameters, high-frequency rTMS (10–20 Hz) or iTBS applied to the left or right DLPFC is current best practice; a minimum of 10 sessions is recommended, with concurrent psychotherapy preferred (47, 48). Absolute contraindications include active seizure disorder, intracranial metallic implants, serious cardiovascular disease, and pregnancy. Key clinical limitations include incomplete protocol standardization and significant cost and accessibility barriers (46, 48).

### Transcranial direct current stimulation

tDCS applies weak electrical currents (1–2 mA) to the scalp, modulating cortical excitability through subthreshold shifts in neuronal membrane potential. Its primary advantages are portability, low cost, and minimal side effects (49). Evidence supports positive effects on craving and use frequency across tobacco, stimulant, and opioid use disorders, with more variable results in alcohol use disorder (46, 49). Bormann et al. (50) found that combining TMS or tDCS with medication-assisted treatment produced significantly greater craving reductions than sham stimulation alone (Hedges’ *g* = −0.42), reflecting a theoretically coherent strategy: pharmacotherapy stabilizes the neurochemical environment while neuromodulation directly targets the prefrontal control deficits that pharmacotherapy alone does not address.

### Deep brain stimulation

DBS involves the surgical implantation of electrodes in subcortical reward circuit structures — primarily the nucleus accumbens (NAc), the subthalamic nucleus (STN), or the dorsal anterior cingulate cortex (dACC) — and is indicated exclusively for severe, treatment-resistant cases in which multiple adequate trials of pharmacotherapy and psychotherapy have failed (51, 52). Small-sample pilot studies have reported reductions in craving and substance use in alcohol and nicotine dependence following NAc-targeted DBS (52). Clinical eligibility requires inadequate response to at least two distinct first-line treatment combinations, comprehensive neuropsychological evaluation, and multidisciplinary team consensus.

### The mechanism of integration: why neuromodulation and psychotherapy work better together

Psychotherapy approaches most evidence-based in addiction — CBT, motivational interviewing, relapse prevention, trauma-focused approaches such as EMDR — all require active prefrontal engagement: monitoring internal states, inhibiting automatic responses, restructuring cognitive appraisals, sustaining goal-directed behavior. These are precisely the executive functions that DLPFC hypofunction impairs. Neuromodulation addresses this constraint directly: by enhancing DLPFC excitability, it creates a neurobiological window of improved prefrontal capacity during which psychotherapeutic learning is more likely to consolidate. Mehta et al. (46) recommend concurrent psychotherapy during active rTMS protocols for precisely this reason. The same logic extends to the integration of neuromodulation with pharmacotherapy and social rehabilitation: each layer enables the others, and none is sufficient alone.

## Discussion

### Conceptualizing addiction beyond the brain circuit

The evidence reviewed in the preceding sections converges on a consistent picture: addiction involves measurable, circuit-level disruptions that unfold across a predictable three-stage cycle, are shaped by genetic vulnerability and epigenetically encoded relational history, and are amenable — at least in part — to targeted biological intervention through neuromodulation. **Table 1** integrates these findings into a clinically oriented summary linking each stage to its primary neurocircuits, neurochemical changes, clinical presentation, and neuromodulation targets.

**Table 1:** The addiction cycle, neurocircuits, clinical features and neuromodulation targets.

Addiction stage	Dominant brain circuits	Neurochemic al processes	Clinical features	Potential neuromodulation targets
Binge/Intoxication	Mesocorticolimbic dopamine system (VTA–NAc–ventral pallidum), ventral striatum.	Increased dopamine release, incentive sensitization, glutamatergic plasticity.	Intense expectation of pleasure, strong approach behavior toward the substance, initially more “goal-directed” use.	Re-regulation of reward expectation via DLPFC (TMS, tDCS), combined with behavioral interventions.
Withdrawal/Negative Affect	Extended amygdala (amygdala, BNST, NAc shell), stress circuits.	Corticotropin-releasing factor (CRF), dynorphin, noradrenergic stress response, anti-reward processes.	Dysphoria, anxiety, irritability, substance use “not to feel good, but to not feel bad” (negative reinforcement).	Prefrontal and limbic modulation targeting stress regulation, strengthening emotion regulation skills with psychotherapy.
Preoccupation/Craving	Prefrontal cortex (DLPFC, vmPFC, OFC), anterior cingulate cortex, insula, dorsal striatum.	Impairment in executive functions, decreased impulse control, increased salience attribution to substance cues.	Compulsive drug seeking, strong craving, tendency to relapse, impaired decision-making.	Strengthening executive function and inhibition with DLPFC-focused TMS/tDCS, combinations of neuromodulation + CBT/motivational interviewing.

VTA,Ventral Tegmental Area; NAc, Nucleus Accumbens; BNST, Bed Nucleus of the Stria Terminalis; DLPFC, Dorsolateral Prefrontal Cortex; vmPFC, Ventromedial Prefrontal Cortex; OFC, Orbitofrontal Cortex; TMS, Transcranial Magnetic Stimulation; tDCS, Transcranial Direct Current Stimulation; CBT, Cognitive Behavioral Therapy (Bilişsel Davranışçı Terapi); CRF, Corticotropin-Releasing Factor.

As **Table 1** illustrates, each stage of the addiction cycle is associated with distinct circuit-level dysfunction and thus with distinct intervention logic. DLPFC-targeted rTMS or tDCS is most directly indicated in the preoccupation/craving stage. The withdrawal/negative affect stage may benefit from iTBS protocols that simultaneously address depressive symptomatology, as well as trauma-informed psychotherapy. The binge/intoxication stage is least directly addressable through neuromodulation alone and requires the full integration of pharmacotherapy, behavioral intervention, and social support. This stage-specific differentiation argues against applying neuromodulation uniformly and in favor of circuit-informed, individualized treatment planning.

### The brain disease model: contribution and critique

The Brain Disease Model of Addiction (BDMA) has made genuine contributions: it shifted discourse from moral failure to medical disorder, reduced stigma in many contexts, and generated a productive neuroscientific research agenda. At the same time, Lewis (as discussed in 53) argues that the neurobiological changes observed in addiction are better understood as deeply entrenched learning processes. Heyman (54, 55) extends this by noting that high rates of natural remission in community samples suggest addiction is sensitive to contextual contingencies in ways a deterministic disease model struggles to accommodate. The neurobiological changes documented in addiction research are real — the question is whether they constitute an irreversible disease or a state of profound, context-sensitive neuroplasticity. The evidence reviewed in this paper supports the latter: the addicted brain is not broken beyond repair; it is a brain that has adapted to specific conditions, and can adapt again under different ones.

### Addiction as a disorder of social connection: the neurobiological case

Galiza Soares et al. (6) demonstrated a bidirectional causal relationship between social deprivation and opioid dependence susceptibility, finding that the endogenous opioid system regulates both substance reward and social bonding. Social disconnection and substance dependence are not parallel problems that happen to co-occur — they share a neurobiological substrate. The epigenetic evidence reinforces this argument: the methylation patterns that confer addiction vulnerability are, in large part, the biological residue of early relational failure (3–5). Sinclair et al. (56) found that social connectedness was the single strongest predictor of sustained recovery. Social rehabilitation is not an adjunct to treatment — it is a neurobiological intervention in its own right.

### Ethical dimensions: autonomy, stigma, and the limits of biological intervention

Three ethical issues merit attention. First, autonomy and consent: addiction, by its neurobiological nature, impairs the very executive functions that informed consent requires. For DBS, multidisciplinary evaluation, longitudinal consent processes, and explicit capacity assessment are ethical necessities (52). Second, stigma and responsibility: framing addiction as a brain disease reduces blame in some contexts but risks replacing moral stigma with a different kind — that of being neurobiologically defective and incapable of change. A framework emphasizing neuroplasticity, recovery, and relational factors may better preserve agency. Third, biological reductionism in clinical practice: when neuromodulation is positioned as primary or standalone, it communicates that addiction is a brain problem requiring a brain fix — sidelining relational, traumatic, and social dimensions central to the evidence and risking iatrogenic passivity.

### Toward a unified position

It is worth being explicit about what is, and is not, being claimed as new here. The proposition that addiction reflects the joint operation of biological, psychological, and social factors is not itself novel; it is a direct extension of Engel’s (1977) biopsychosocial model and, more recently, of RDoC-based dimensional frameworks for psychiatric disorders (8). What this review contributes is not a new general theory of addiction, but a specific operationalization of that general theory: a stage-specific mapping (**Table 1**) that assigns each neurocircuit stage of the addiction cycle to a corresponding neuromodulation target, and that situates this biological layer within a broader psychotherapeutic and social-relational framework elaborated in the sections above, treating the three layers as bidirectionally interacting rather than hierarchically ordered. The conceptual debates surveyed in this section — brain disease versus learning disorder, neurobiological versus psychosocial — are more productively understood as complementary perspectives on a genuinely complex phenomenon. The three-layer integrative framework proposed in this review does not resolve these debates by declaring one side correct; it integrates them by assigning each perspective its appropriate domain of explanatory authority. Neuromodulation is most valuable when it serves the larger process of recovery: creating the neurobiological conditions under which psychotherapy can be effective and social rehabilitation can take hold.

## Conclusion and clinical recommendations

This integrative review set out to argue that addiction is not solely a brain circuit disorder, but also a disorder of social connection and meaning — and that effective treatment must reflect this complexity. The evidence reviewed across neurobiological, epigenetic, and psychosocial domains consistently supports this argument, while also affirming that neurobiological intervention remains an indispensable component of comprehensive care.

### What the evidence establishes

The three-stage neurocircuit model provides a mechanistically coherent account of why addiction is compulsive, chronic, and resistant to change through conscious self-control alone -– that is, resistant to the assumption that motivation or intention, unsupported by circuit-level intervention, is sufficient to reverse compulsive use (1, 14). Neuroimaging evidence confirms these circuit disruptions directly in living human brains (15, 16, 19). Genetic and epigenetic findings establish that neurobiological vulnerabilities are shaped by a polygenic architecture (24, 25) and by epigenetic mechanisms through which early adversity leaves lasting imprints on the same circuits that addiction subsequently dysregulates (3, 4). Neuromodulation techniques — particularly rTMS and tDCS targeting the DLPFC — have demonstrated moderate-to-high effect sizes in reducing craving, with the strongest evidence supporting multi-session protocols combined with psychotherapy or pharmacotherapy (46, 50). These two lines of evidence converge on distinct but complementary circuits: rTMS and tDCS act directly on the DLPFC to restore top-down executive control over craving, whereas social connection operates through the endogenous opioid system to regulate reward and stress reactivity from a different neurobiological entry point (6) — a convergence increasingly recognized in systematic reviews that integrate neurobiological findings with psychotherapeutic evidence for addiction, including CBT, EMDR, and mindfulness-based relapse prevention (57). Finally, social connectedness is the strongest single predictor of sustained recovery (56); social isolation shares a neurobiological substrate with opioid dependence (6); and because this substrate is neurobiological rather than merely motivational, social rehabilitation should be understood as a neurobiological intervention in its own right, not an adjunct to biological treatment.

### A three-layer integrative treatment framework

The central clinical contribution of this review is a three-layer integrative treatment framework in which each layer creates conditions that enable the others to work.

*Layer 1 — Biological intervention.* Pharmacotherapy and neuromodulation address circuit-level disruptions. Medication remains the first-line biological intervention for many substance use disorders and should not be treated as secondary to neuromodulation. For opioid use disorder, methadone achieves somewhat better treatment retention than buprenorphine (12-month retention around 61% versus 45% across a synthesis of more than one million patients), while buprenorphine is associated with fewer overdose-related hospitalizations, particularly among people using more than one substance (58, 59). For alcohol use disorder, naltrexone and acamprosate are the best-supported first-line medications, each producing a real but modest reduction in return-to-drinking rates (60).

Disulfiram, which produces an aversive reaction upon alcohol consumption through inhibition of aldehyde dehydrogenase, remains a third FDA-approved option; in a clinical cohort of patients with severe AUD, disulfiram used as an adjunct to addiction-focused treatment was associated with continuous abstinence in half of patients at one year, underscoring its continued clinical relevance despite a scarcity of high-quality long-term randomized trials (61). Pharmacotherapy for other substances shows a similar reliance on a narrower evidence base than AUD: nicotine use disorder is comparatively well-served, with varenicline and combination nicotine replacement therapy demonstrating the highest efficacy among first-line agents (62), whereas cannabis and cocaine use disorders still lack any FDA-approved medication, leaving clinicians reliant on off-label, typically combination-based strategies. This pattern is consistent with a broader finding across substance use disorders: because addiction involves multiple simultaneously dysregulated pathways, enzymes, neurotransmitters, and receptors, monotherapy — while superior to no treatment — is consistently outperformed by combination pharmacological approaches (63). This asymmetry across substances is itself clinically relevant: for cannabis and cocaine use disorders specifically, where no approved pharmacotherapy exists at all, neuromodulation represents one of the few available evidence-based biological options, rather than a supplementary add-on to an already-effective first-line medication. These medications work best alongside counseling rather than in place of it: a review focused on people with co-occurring PTSD and substance use found that medication alone had only a small effect on drug use outcomes, reinforcing this review’s broader argument that pharmacotherapy is one part of a larger picture rather than a stand-alone solution, especially for people with a trauma history (64). In practice, access to these medications remains limited by stigma, provider shortages, and geographic barriers (65). Neuromodulation faces a distinct and, in most healthcare settings, more severe set of access barriers: it requires specialized equipment, trained personnel, and multi-session protocols that are largely unavailable outside well-resourced urban treatment centers (46, 48). Rather than positioning one biological intervention as a solution to the access limitations of another, this review’s three-layer framework treats differential accessibility as a structural constraint that must be addressed through health-system-level investment, alongside — not instead of — the clinical integration proposed here. Beyond medication, neuromodulation — specifically rTMS with high-frequency or iTBS protocols targeting the left or right DLPFC — is most indicated for individuals with high craving burden, documented DLPFC hypofunction, significant impulsivity, and inadequate response to first-line treatments (46). tDCS offers a portable, lower-cost alternative for outpatient settings. DBS remains reserved for severe, treatment-resistant cases following multidisciplinary evaluation.

*Layer 2 — Psychological intervention.* Trauma-informed approaches — EMDR and trauma-informed CBT — are the primary psychological intervention for individuals with high ACE burden, targeting the escape and avoidance functions underlying substance use, not merely craving management (3, 4). Evidence for the general efficacy of psychological approaches in substance use disorders is real but modest: a meta-review synthesizing 23 meta-analyses (78 effect sizes) found predominantly low-to-moderate quality evidence, with small-to-moderate effects for cognitive-behavioral therapy, motivational approaches, and contingency management relative to inactive controls (66). Consistent with this modest-but-real effect, a randomized trial adding brief CBT to methadone maintenance therapy improved anxiety and insomnia but not depression or social functioning, underscoring that psychological intervention functions as one component of a combined protocol rather than a stand-alone cure (67). Delivering this layer competently is itself a specialized skill set: a systematic review underpinning a national addictions counselling competency framework identifies trauma-informed formulation, risk management, and relational skill as distinct from generic psychotherapy training (68). The critical mechanism is timing: neuromodulation enhances prefrontal capacity, creating a neurobiological window during which psychotherapeutic learning is more likely to consolidate. The precise duration of this window, and the degree to which its therapeutic value decays as the interval between stimulation and psychotherapy lengthens, has not been systematically established and remains an important open question for protocol design. In the absence of definitive dose–timing data, the pragmatic recommendation advanced here is that psychological intervention be delivered concurrently with or as soon as feasible after neuromodulation sessions, on the assumption -– consistent with general principles of activity-dependent plasticity -– that a shorter interval preserves more of the window’s benefit than a longer one.

*Layer 3 — Social and relational intervention.* Given that social connectedness is the strongest predictor of recovery and that social isolation operates through the same opioid circuitry as substance dependence, social rehabilitation must be systematically embedded in treatment. Family counseling, peer support groups, and social skills development directly address the relational deficits that epigenetic research identifies as central to addiction vulnerability (36, 56). Empirically, perceived social support predicts lower relapse risk independent of childhood trauma history (69), and structural equation modeling in a compulsory-rehabilitation sample shows that social support increases motivation to quit both directly and indirectly, by reducing perceived stress and addiction severity (S. 45). Attachment-focused therapeutic approaches are not soft additions to a biomedical protocol — they are the relational layer through which biological recovery becomes sustainable, though the evidence base counsels realism about their relative weight: in a randomized comparison with adolescents, attachment-based therapy produced smaller effect sizes than acceptance- or emotion-focused approaches, suggesting it is best positioned as a complementary rather than first-line intervention for relational deficits (70). Labeling Layer 3 “neurobiological” requires a clarification of measurement: unlike Layer 1, where DLPFC-targeted neuroimaging parameters are readily available, or Layer 2, where psychometric reduction in trauma symptoms serves as a proxy outcome, Layer 3 currently lacks a routinely used biomarker of its own. Candidate indices — such as opioid-system-related neuroimaging or endocrine markers of social stress buffering (e.g., cortisol reactivity) — would need to be validated longitudinally before they could confirm that a social intervention has produced a genuinely neurobiological, rather than purely behavioral or subjective, effect. Finally, although the layers are presented here in the order 1→2→3 for clarity of exposition, this sequence is not intended as a fixed clinical priority: the framework is conceived as bidirectional, such that, for example, strong pre-existing social support (Layer 3) can increase a patient’s responsiveness to psychotherapy (Layer 2), just as psychotherapy can increase engagement with social rehabilitation, and biological readiness (Layer 1) can be enhanced by either.

### Limitations and future research priorities

As an integrative review, this paper synthesizes findings across heterogeneous methodologies without applying systematic meta-analytic inclusion/exclusion procedures. The epigenetic literature is predominantly cross-sectional, limiting causal inference. Neuromodulation evidence remains limited by incomplete protocol standardization and insufficient long-term follow-up data. Future research should prioritize: (1) multicenter randomized controlled trials testing integrated biological-psychological-social protocols with follow-up of at least 12–24 months; (2) longitudinal epigenetic studies to establish state versus trait methylation signatures; (3) neuroimaging-guided neuromodulation protocols that individualize stimulation targets; (4) investigation of the epigenetic effects of psychotherapy and social rehabilitation; and (5) cost-effectiveness and implementation analyses for integrated protocols within existing treatment systems (4, 25, 46).

### Closing reflection

The neuroscience of addiction tells us that the addicted brain is not broken — it is a brain that has adapted, with extraordinary efficiency, to conditions of pain, disconnection, and deprivation. The epigenetic evidence tells us that much of that adaptation began before the first substance was ever used. The relational evidence tells us that recovery is fundamentally a process of reconnection: to safety, to others, and to a life worth living without chemical escape. A treatment framework worthy of this complexity does not choose between biology and relationship, between the brain and the person. It holds both.
